# Assessment of the Efficacy and Clinical Utility of Different Circulating Tumor Cell (CTC) Detection Assays in Patients with Chemotherapy-Naïve Advanced or Metastatic Non-Small Cell Lung Cancer (NSCLC)

**DOI:** 10.3390/ijms22020925

**Published:** 2021-01-18

**Authors:** Maria A. Papadaki, Ippokratis Messaritakis, Oraianthi Fiste, John Souglakos, Eleni Politaki, Athanasios Kotsakis, Vassilis Georgoulias, Dimitrios Mavroudis, Sofia Agelaki

**Affiliations:** 1 Laboratory of Translational Oncology, School of Medicine, University of Crete, Heraklion, Vassilika Vouton, 71110 Crete, Greece; papadaki_maria1@yahoo.gr (M.A.P.); imessar@edu.med.uoc.gr (I.M.); johnsougl@gmail.com (J.S.); lenapolitaki@yahoo.gr (E.P.); mavroudis@uoc.gr (D.M.); 2 Department of Clinical Therapeutics, School of Medicine, National and Kapodistrian University of Athens, 11528 Athens, Greece; ofiste@med.uoa.gr; 3 Department of Medical Oncology, University General Hospital of Heraklion, Vassilika Vouton, 71110 Crete, Greece; 4 Department of Oncology, University General Hospital of Larissa, School of Health Sciences, University of Thessaly, 413 34 Larissa, Greece; thankotsakis@hotmail.com; 5 Department of Medical Oncology, IASO General Hospital, 15562 Athens, Greece; georgouliasv@gmail.com

**Keywords:** liquid biopsy, circulating tumors cells, non-small cell lung cancer, CellSearch system, CEACAM5mRNA, prognostic biomarkers, predictive biomarkers, first-line chemotherapy, monitoring

## Abstract

We herein investigated the detection frequency and clinical relevance of circulating tumor cells (CTCs) in chemotherapy-naïve stage IIIB/IV non-small cell lung cancer (NSCLC), by using the CellSearch and real-time CEACAM5mRNA assays. Blood samples from 43 patients were obtained at different time points during first-line chemotherapy. CellSearch revealed the detection of ≥1 CTCs in 41.9%, 40.9%, and 16.7% of patients at baseline, post-1st, and post-2nd treatment cycle, respectively, and of ≥5 CTCs in 11.6%, 9.1%, and 5.6%, respectively. CEACAM5mRNA+ CTCs were detected in 29.3% and 16% of patients pre- and post-treatment, respectively. The positivity concordance between the two assays was 2.2%. CTC-detection by CellSearch (≥5 CTCs: *p* = 0.004), CEACAM5mRNA (*p* = 0.010), or by any assay (*p* = 0.000) was associated with disease progression. Reduced survival was demonstrated for patients harboring ≥5 CTCs (progression-free survival; PFS: *p* = 0.000; overall survival; OS: *p* = 0.009), CEACAM5mRNA+ CTCs (PFS: *p* = 0.043; OS: *p* = 0.039), and CTCs by any assay (PFS: *p* = 0.005; OS: *p* = 0.006, respectively). CTC-detection by any assay independently predicted for increased risk of relapse (hazard ratio; HR: 3.496; *p* = 0.001) and death (HR: 2.866; *p* = 0.008). CellSearch-positivity either pre-, post-1st, or post-2nd cycle, was predictive for shorter PFS (*p* = 0.036) compared to negativity in all time points. Persistent CEACAM5mRNA-positivity pre- and post-treatment was associated with reduced PFS (*p* = 0.036) and OS (*p* = 0.026). In conclusion, CTC detection and monitoring using the CellSearch and CEACAM5mRNA assays provides valuable and complementary clinical information for chemo-naïve advanced or metastatic NSCLC.

## 1. Introduction

Non-small cell lung cancer (NSCLC) accounts for 85% of lung cancers, and despite the recent advances in therapeutic modalities, including molecularly targeted therapies and immunotherapy, patient prognosis remains dismal [1]. Taking into consideration that almost 80% of newly diagnosed cases, annually, will eventually succumb [2], there is a highly unmet need for a reliable and accurate prognostic biomarker, to rationally guide clinical decisions.

Circulating tumor cells (CTCs) are detected in peripheral blood (PB) of patients with solid tumors, and include the cell subsets that participate in the development of distant metastases [3]. CTC evaluation represents a liquid biopsy tool that could serve as a minimally invasive substitute for conventional biopsies, offering the potential for early detection of disease recurrence, real-time assessment of treatment response, and the detection of treatment-induced genomic alterations [4,5,6]. In NSCLC, there is only limited data on the longitudinal monitoring of CTCs during treatment, whereas the potential prognostic benefit it may add to the baseline CTCs status needs further evaluation [7,8,9].

Despite the great availability of sensitive and specific CTC detection methodologies, the detection rates and CTC numbers obtained by different assays vary significantly, even at the individual patient level [10,11,12,13]. Consequently, there is still no consensus on a universal CTC detection assay suitable for routine implementation in clinical practice. The semi-automated CellSearch system (Menarini Silicon Biosystems), which combines an epithelial cell adhesion molecule (EpCAM)-based CTC enrichment with a cytokeratin-based CTC detection, is the only U.S. Food and Drug Administration (FDA) approved assay for the enumeration of CTCs as a prognostic biomarker in breast, prostate, and colorectal cancer [14], but has not been approved for use in NSCLC patients. Although CellSearch holds significant prognostic and predictive implications in NSCLC, the detection frequency in this cancer entity is rather low, and different thresholds have been used to define patient positivity [7,15,16]. 

On the other hand, quantitative reverse transcription-PCR (RT-qPCR) has been widely used for the detection and quantification of distinct CTC markers in PB of cancer patients [17]. In NSCLC, the mRNA expression of cytokeratin 19 (CK19) is the most well-studied marker for CTCs; in this context, our group has previously demonstrated the clinical value of the detection and monitoring of CK19mRNA+ CTCs in locally advanced or metastatic NSCLC [18]. The mRNA expression of carcinoembryonic antigen-related cell adhesion molecule 5 (CEACAM5) has been also assessed in PB of patients with NSCLC [19,20,21]; however, contradictory results were shown regarding the detection frequency and clinical relevance of *CEACAM5*mRNA+ CTCs, whereas their kinetics during treatment has not been investigated so far. It should be noted that the utility of *CEACAM5*mRNA expression as a CTC marker has raised significant doubts, due to its detection in healthy individuals [22] and patients with inflammatory bowel disease [23]. To overcome this limitation, our group established a new RT-qPCR assay that allocates the specific detection of *CEACAM5*, excluding the amplification of the *CEACAM1* splicing variant, which is also expressed by normal white blood cells [24]. The high specificity, repeatability, and reproducibility of our method were confirmed in two studies in early [25] and metastatic [26] colorectal cancer, further showing the adverse prognostic relevance of *CEACAM5*mRNA+ CTCs in both settings.

In the current study, we investigated the presence of CTCs in patients with chemo-naïve advanced or metastatic NSCLC, by using a specific RT-qPCR assay for the detection of *CEACAM5*mRNA+ CTCs, along with the FDA-approved CellSearch system. We aimed: a) to compare the analytical performance of the two detection methods, b) to evaluate the kinetics of CTCs during first-line chemotherapy, and c) to estimate the clinical value of the detection and monitoring of CTCs by using either assay or their combination.

## 2. Results

### 2.1. Patient and Disease Characteristics

The current study included 43 patients with chemo-naïve advanced and/or metastatic NSCLC who received first-line chemotherapy. Patient and disease characteristics are listed in Table 1. Briefly, there were 39 (90.7%) men and 4 (9.3%) women, with a median age of 67 years (range 46–86 years). The majority of patients had metastatic disease at the time of diagnosis (81.4%). Therapy consisted of platinum-based doublet chemotherapy in 88.4% of patients; combinations included taxanes (30.23%), pemetrexed (27.91%), gemcitabine (25.58%), or vinorelbine (4.65%). Docetaxel monotherapy was administered in 11.6% of patients. At the time of the analysis, disease progression and death were recorded in 39 (90.7%) and 32 (74.4%) patients, respectively. Median progression-free survival (PFS) and overall survival (OS) was 5.80 months (95% CI: 4.47–7.13) and 12.37 months (7.78–16.95), respectively. 

### 2.2. CTC Detection at Baseline (Before First-line Chemotherapy) by Using Different Assays

**Figure 1:**
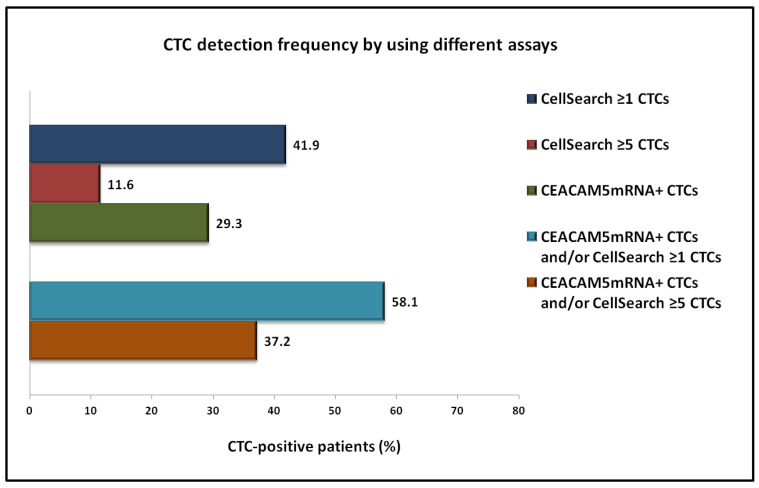
Frequency of CTC detection in patients with NSCLC at baseline of first-line chemotherapy, by using the CellSearch or the *CEACAM5*mRNA assay, or their combination.

CellSearch analysis revealed the detection of ≥1 CTCs and ≥5 CTCs among 18 (41.9%) and 5 (11.6%) out of 43 patients at baseline (Figure 1). A total of 192 CTCs were detected, with a mean number of 4.47 CTCs/patient (range: 0–108).

The RT-PCR assay revealed the identification of *CEACAM5*mRNA+ CTCs in 12 out of 41 patients (29.3%) at baseline (Figure 1). Combined analysis revealed the detection of CTCs by any method in 58.1% and in 37.2% of patients, when using the threshold of ≥1 and ≥5 CTCs, respectively, for CellSearch positivity (Figure 1).

No correlation was observed between the number of *CEACAM5*mRNA+ CTCs and CTCs detected using CellSearch (*p* = 0.945; Spearman's rho analysis). Additionally, there was no correlation regarding the status of patients among the two detection assays; any concordance observed was mostly related to the negative rather than the positive cases (Table 2).

### 2.3. Monitoring of CTCs during First-Line Chemotherapy 

#### 2.3.1. Using the CellSearch System

**Figure 2:**
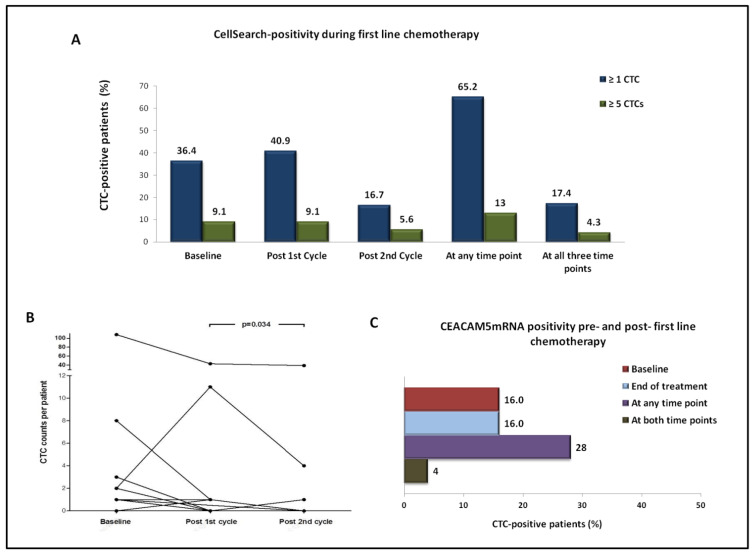
Kinetics of CTCs during first-line chemotherapy by using different detection approaches. (**A)** Percentage of patients with detectable CTCs by CellSearch at different time points, using either ≥1 CTCs or ≥5 CTCs as a threshold for positivity. (**B)** Per patient distribution of CTCs detected using CellSearch at different time points. (**C)** Percentage of patients with detectable CTCs using the *CEACAM5*mRNA assay at the start and end of treatment**.**

CellSearch analysis after the administration of the first cycle of treatment revealed the detection of ≥1 CTCs and ≥5 CTCs in 9 (40.9%) and 2 (9.1%) out of 22 patients, respectively (Figure 2A). The number of CTCs increased, decreased, or remained unchanged after the first cycle of treatment, in 7, 6, and 9 out of 22 patients, respectively (Figure 2B).

Following two cycles of treatment, ≥1 CTCs and ≥5 CTCs were detected in 3 (16.7%) and 1 (5.6%) out of 18 patients, respectively (Figure 2A). CTC counts increased, decreased, or remained unchanged between baseline and the second cycle of treatment, in 1, 5, and 12 out of 18 patients, respectively (Figure 2B).

In 65.2% of patients, ≥1 CTCs were detected at any time point, and in 17.4% at all three time points. In accordance, ≥5 CTCs were detected at any time point in 13% of patients, whereas one patient harbored ≥5 CTCs at all time points (Figure 2A).

At the CTC level, CTC counts significantly decreased between the first and second treatment cycle (mean no of CTCs/patient: 3.47 vs. 2.44, respectively, *p* = 0.034; Wilcoxon) (Figure 2B); a numerical decrease in CTC counts was demonstrated between baseline and post-1st cycle (mean no of CTCs/patient: 5.73 vs. 2.82, respectively, *p* = 0.641; Wilcoxon test), as well as between baseline and post-2nd cycle of treatment (mean no of CTCs/patient: 6.50 vs. 2.44, respectively, *p* = 0.168; Wilcoxon).

#### 2.3.2. Using Real-Time RT-PCR for CEACAM5mRNA

*CEACAM5*mRNA+ CTCs were identified in 4/25 (16%) patients at baseline, as well as in 4/25 (16%) post-treatment. Seven patients (28%) deemed CTC-positive at any time point, whereas 18 (72%) were CTC-negative at both time points; one patient (4%) remained CTC-positive at both time points (Figure 2C).

The number of *CEACAM5*mRNA+ CTCs increased, decreased, or remained unchanged, in 5, 3, and 17 out of 25 patients, respectively. There was no significant change in the number of *CEACAM5*mRNA+ CTCs between the start and the end of treatment (mean: 0.19 vs. 0.22, respectively, *p* = 0.889; Wilcoxon t test).

### 2.4. Correlation of the Detection and Kinetics of CTCs with Clinicopathological Data and Response to Treatment

Using CellSearch, the detection of ≥1 CTCs or ≥5 CTCs at baseline was associated with metastases in two or more sites (*p* = 0.002 and *p* = 0.021, respectively; Chi-square test). The detection of ≥1 CTCs was also associated with the presence of bone metastases (*p* = 0.035; Chi-square test). No association was shown between clinicopathological characteristics and the baseline detection of *CEACAM5*mRNA+ CTCs or CTC detection by any assay. Additionally, there was no association between clinicopathological characteristics and the status or kinetics of CTCs during treatment, by using either CellSearch or the *CEACAM5*mRNA assay.

Regarding the response to treatment at first evaluation, there was a correlation between the baseline detection of ≥5 CTCs by CellSearch or of *CEACAM5*mRNA+ CTCs and progressive disease (PD) (*p* = 0.004 and *p* = 0.010, respectively; Chi-square test) (Table 3). The predictive value of CellSearch was significantly improved when combined with the *CEACAM5*mRNA assay; CTC detection by any method was associated with PD (*p* = 0.002 and *p* = 0.000 when using the CellSearch cut-offs of ≥1 and ≥5 CTCs, respectively (Table 3). No other associations were observed between response rates and the status or kinetics of CTCs during treatment, by the use of either methodology.

### 2.5. Prognostic Relevance of the Detection of CTCs at Baseline of First-Line Chemotherapy

**Figure 3:**
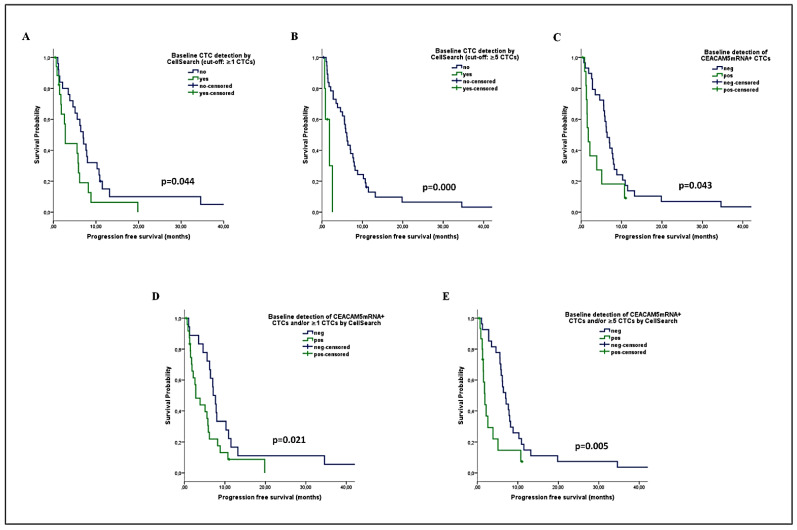
Progression-free survival (PFS) analysis among patients with NSCLC undergoing first-line chemotherapy, according to the baseline detection of CTCs by using the CellSearch or the *CEACAM5*mRNA assay (**A**–**C**) or by their combination (**D**,**E**).

Kaplan Meier analysis revealed a significantly reduced PFS among patients harboring ≥1 CTCs (median PFS: 2.8 vs. 7 months; *p* = 0.044) and especially ≥5 CTCs (median PFS: 1.9 vs. 6.2 months; *p* = 0.000) by CellSearch (Figure 3A,B). The detection of *CEACAM5*mRNA+ CTCs was also associated with lower PFS (median: 1.8 vs. 6.4 months; *p* = 0.043) (Figure 3C). The combined evaluation of CTCs improved their prognostic value; a shorter PFS was recorded for patients with *CEACAM5*mRNA+ CTCs and/or ≥1 CTCs by CellSearch (median: 2.8 vs. 7.1 months; *p* = 0.036), as well as for those harboring *CEACAM5*mRNA+ CTCs and/or ≥5 CTCs (median: 1.9 vs. 7 months; *p* = 0.005) Figure 3D,E).

**Figure 4:**
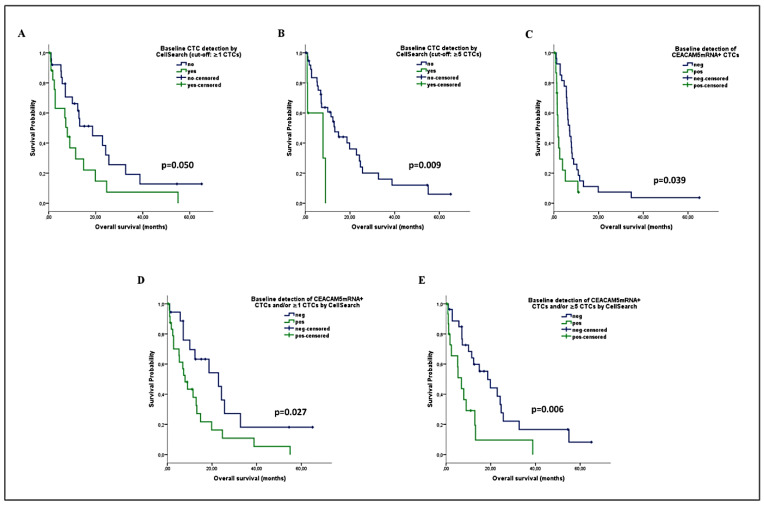
Overall survival (OS) analysis among patients with NSCLC undergoing first-line chemotherapy, according to the baseline detection of CTCs by using the CellSearch or the *CEACAM5*mRNA assay alone (**A**–**C**) or in combination (**D**,**E**).

In accordance, a reduced OS was demonstrated among patients with ≥1 CTCs (median: 7.9 vs. 18.7 months; *p* = 0.050) or ≥5 CTCs (median: 7.9 vs. 13.2 months; *p* = 0.009) by CellSearch, or with *CEACAM5*mRNA+ CTCs (median: 5.4 vs. 14.9 months; *p* = 0.039) (Figure 4A–C). The combined detection of CTCs significantly improved their prognostic relevance in predicting OS (Figure 4D,E), by using either ≥1 CTCs (median: 7.9 vs. 22.9 months; *p* = 0.027) or ≥5 CTCs (median: 6.9 vs. 18.7 months; *p* = 0.006) as a threshold for CellSearch positivity.

In the Univariate Cox regression analysis for PFS, an increased risk for relapse was demonstrated for patients harboring ≥1 CTCs by CellSearch (HR: 1.941; *p* = 0.048) or ≥5 CTCs by CellSearch (hazard ratio; HR: 6.948; *p* = 0.002), or *CEACAM5*mRNA+ CTCs (HR: 2.134; *p* = 0.048) at baseline, and especially for patients with *CEACAM5*mRNA+ CTCs and/or ≥1 CTCs (HR: 1.996; *p* = 0.039), and those with *CEACAM5*mRNA+ CTCs and/or ≥5 CTCs (HR: 2.672; *p* = 0.006) (Table 3). Multivariate analysis confirmed that the presence of bone metastases (HR: 3.017; *p* = 0.005) and the detection of ≥5 CTCs by CellSearch (HR: 3.810, *p* = 0.034) or of *CEACAM5*mRNA+ CTCs (HR: 3.330, *p* = 0.004), and especially the combined detection of *CEACAM5*mRNA+ CTCs and/or ≥5 CTCs (HR: 3.496; *p* = 0.001), were independent factors predicting for an increased risk of relapse (Table 3).

Accordingly, the Univariate Cox regression analysis for OS revealed that the detection of ≥5 CTCs (HR: 4.026; *p* = 0.015) or *CEACAM5*mRNA+ CTCs (HR: 2.188; *p* = 0.045), as well as the combined detection of *CEACAM5*mRNA+ CTCs and/or ≥1 CTCs (HR: 2.242; *p* = 0.032), and especially of *CEACAM5*mRNA+ CTCs and/or ≥5 CTCs (HR: 2.713; *p* = 0.008), predicted for increased risk of death (Table 3). In the multivariate analysis, performance status (PS) (HR: 5.014; *p* = 0.002), the presence of bone metastases (HR: 3.257; *p* = 0.003), the detection of *CEACAM5*mRNA+ CTCs (HR: 2.716, *p* = 0.017), and especially the combined detection of *CEACAM5*mRNA+ CTCs and/or ≥5 CTCs by CellSearch (HR: 2.866; *p* = 0.008), were independent factors predicting for high risk of death (Table 3).

### 2.6. Prognostic Relevance of the Kinetics of CTCs during First-Line Chemotherapy

**Figure 5:**
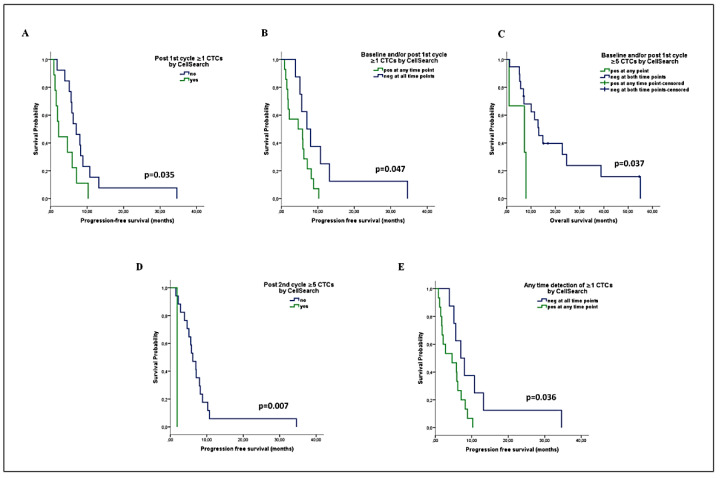
Prognostic relevance of the kinetics of CTCs detected by CellSearch during first-line chemotherapy in patients with advanced NSCLC. (**A**) PFS analysis according to CTC detection post-1st cycle of treatment, (**B**,**C**) PFS and OS rates according to the combined CTC status at baseline and/or post-1st cycle of treatment, (**D**) PFS analysis according to CTC detection post-2nd cycle, (**E**) PFS according to the combined CTC status at any of the three time points among baseline, post-1st cycle, and post-2nd cycle.

CellSearch analysis after the first cycle of treatment revealed that patients harboring ≥1 CTCs had decreased PFS (median: 1.8 vs. 5.8 months, *p* = 0.035) (Figure 5A). Additionally, a reduced PFS was demonstrated for patients with ≥1 CTCs either at baseline or post-1st cycle, compared to those who remained CTC-negative at both time points (median: 4.6 vs. 7 months, *p* = 0.047) (Figure 5B). In addition, a shorter OS was recorded for patients with ≥5 CTCs either at baseline or post-1st cycle (median: 7.3 vs. 13.2 months, *p* = 0.037) (Figure 5C). No association was observed between the dynamic change in CTC counts (increase, decrease, or unchanged) after the first treatment cycle and survival.

After the second cycle of treatment, the detection of ≥5 CTCs was associated with shorter PFS (median: 1.9 vs. 6.2 mo, *p* = 0.007); however, only one patient harbored ≥5 CTCs (Figure 5D). No other associations were observed regarding the kinetics of CTC counts or the change in CTC status after two cycles of treatment and survival.

The parallel assessment of CellSearch CTC detection at baseline, post-1st and post-2nd cycle of treatment, revealed a shorter PFS among patients who harbored ≥1 CTCs at any of the three time points compared to persistently negative patients (median: 4.6 vs. 7 months, *p* = 0.036) (Figure 5E).

**Figure 6:**
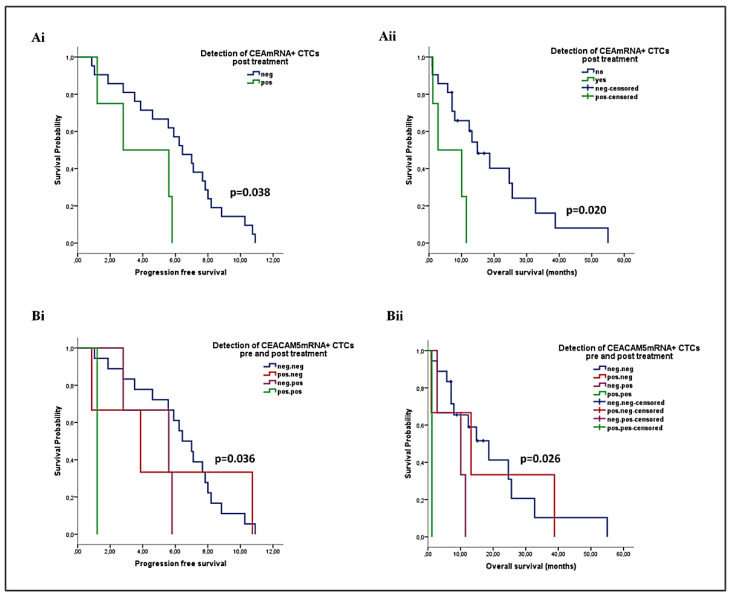
Prognostic relevance of the kinetics of *CEACAM5*mRNA+ CTCs detected by RT-PCR during first-line chemotherapy in patients with advanced NSCLC (**Ai**, **Aii**) PFS and OS rates according to the CTC status at the end of treatment, (**Bi, Bii**) PFS and OS rates according to the combined CTC status pre- and post-treatment.

Regarding *CEACAM5*mRNA+ CTCs, their detection at the end of chemotherapy was predictive for shorter PFS (median: 2.8 vs. 6.4 months; *p* = 0.038) and OS (median: 2.8 vs. 14.9 months; *p* = 0.020) (Figure 6Ai-Aii). Moreover, reduced PFS and OS rates were recorded among patients harboring *CEACAM5*mRNA+ CTCs at both time points, compared to those with CTCs either pre- or post-chemotherapy, and especially those who remained CTC-negative at both time points (median PFS: 1.2 vs. 3.9 vs. 5.6 vs. 6.4 months; *p* = 0.036, and median OS: 1.2 vs. 10.1 vs. 13.2 vs. 18.7; *p* = 0.026) (Figure 6Bi-Bii).

## 3. Discussion

CTCs represent a promising biomarker for NSCLC; however, their detection frequency is low compared to other cancers. Therefore, considerable efforts are underway to identify sensitive and specific CTC detection approaches to be employed in this setting. We herein used the FDA-approved CellSearch platform, and a specific molecular *CEACAM5*mRNA assay for CTC detection, in order to compare their efficacy and clinical significance in patients with chemo-naïve advanced or metastatic NSCLC. We show that CTCs can be frequently detected by using either assay; however, low positivity concordance was demonstrated in individual patients. Importantly, both the CellSearch and *CEACAM5*mRNA assays provide significant prognostic and predictive information, which is substantially improved by complementing the two methods. In addition, the monitoring of CTCs during first-line chemotherapy was also predictive for patients’ clinical outcome.

Numerous studies have shown the prognostic relevance of CTC detection using the CellSearch system in NSCLC; however, different thresholds have been used to define patient positivity [27]. In the current study, we demonstrate the detection of ≥1 CTCs and ≥5 CTCs in 41.9% and 11.6% of patients, respectively, in agreement with previous studies using the above cut-off values [15,16,28]. We further show that the detection of ≥1 CTCs is marginally correlated with reduced PFS and an increased risk for relapse, while the detection of ≥5 CTCs is a strong factor predicting for PD at the first evaluation of response to treatment, for reduced PFS and OS, as well as for increased risk of relapse and death. The weak prognostic relevance obtained using the threshold of 1 CTC indicates a lower specificity, which is further supported by the previously reported detection of one CTC in healthy individuals [14]. The results presented here suggest that, despite the low CTC positivity obtained by the threshold of ≥5 CTCs, it could be used to optimize the stratification of patients with stage IIB/IV NSCLC.

We also investigated the *CEACAM5*mRNA expression in paired PB samples, by using a previously established RT-PCR assay showing high specificity in colon cancer patients [25,26]. In the current study, *CEACAM5*mRNA+ CTCs were detected at baseline in about one-third of treatment-naïve patients, and their detection was associated with PD at first response evaluation, as well as with shorter PFS and OS. Importantly, *CEACAM5*mRNA+ CTCs emerged as an independent factor predicting for increased risk of relapse and death. In a study by Arrieta et al., *CEACAM5*mRNA expression in PB of patients with stage IIIB/IV NSCLC was also associated with poor PFS and OS, but was not predictive of response to first-line chemotherapy [19]. Other studies investigating the presence of *CEACAM5*mRNA+ CTCs in NSCLC included either low numbers of patients [20] or patients at different disease stages [21] and failed to show any correlations with patient outcome.

In the current study, we compared for the first time the detection efficacy and clinical utility of the CellSearch and *CEACAM5*mRNA assays in individual patients. The detection rate obtained by *CEACAM5*mRNA expression was 29.3%, as compared to 41.9% and 11.6% by CellSearch using the thresholds of ≥1 and ≥5 CTCs, respectively. However, no concordance for positivity was observed among the two assays, and as a consequence, positivity rates increased to 58.1% and 37.2%, respectively, when CTCs were in parallel assessed by the two assays. More importantly, the two methods were shown to provide complementary prognostic and predictive information. Although the detection of ≥1 CTCs by CellSearch was marginally associated with clinical outcome, its parallel assessment with *CEACAM5*mRNA expression revealed a strong association with PD, reduced PFS and OS, as well as increased risk for relapse and death. Accordingly, the parallel assessment of ≥5 CTCs by CellSearch and/or *CEACAM5*mRNA+ CTCs revealed the most significant associations with PD, reduced PFS and OS, and emerged as a strong and independent factor associated with increased risk of relapse and death. These findings are in line with previous studies highlighting the necessity of the combined use of multiple markers in order to improve the sensitivity of CTC detection in NSCLC, which might be attributed to the high heterogeneity of CTCs identified in lung cancer [19,21]. In a recent comparative study from our group, different manual and automated enrichment approaches were shown to provide divergent CTC detection rates, and complementary clinical information for patients with advanced NSCLC treated with immunotherapy [29]. Moreover, a discordance between CellSearch and different detection approaches has been extensively reported in NSCLC [11,30]. In the present study, CellSearch was directly compared to the molecular assessment of *CEACAM5* in PB of individual patients, providing first evidence for their complementary role in improving prognostication in NSCLC.

We also evaluated the CTC status of patients at different time points during first-line chemotherapy. Although it is more critical for a prognostic biomarker to be available at baseline, CTCs detected during and/or after treatment probably represent chemo-resistant CTC populations, which may contribute to the real time monitoring of the disease status. In the majority of patients (62.5%), ≥1 CTCs were detected using CellSearch in at least one of the three time points (baseline, post-1st, and post-2nd cycle of treatment), and this finding was clearly associated with reduced PFS, thus increasing the prognostic significance of the baseline CTC detection. Previous studies using the CellSearch system also reported that the post-treatment CTC detection, or the decrease in CTC counts during treatment, may improve prognostication in NSCLC compared to the baseline CTC status [7,8,16], while other studies failed to confirm this observation [31]. On the other hand, the detection of *CEACAM5*mRNA+ CTCs is for the first time evaluated in parallel at baseline and at the end of first-line chemotherapy. The results presented here suggest that the detection of *CEACAM5*mRNA+ CTCs at the end of treatment, as well as the combined CTC assessment at both time points, is highly prognostic for reduced OS. Consequently, monitoring of *CEACAM5*mRNA+ CTCs could more efficiently discriminate the population of patients with poor outcomes, compared to baseline assessment only. 

The current study included a well-defined population of patients with chemo-naïve, stage IIIB/IV NSCLC, which allowed the identification of prognostic biomarkers with clinical relevance. Moreover, the assay used to detect *CEACAM5*mRNA has previously illustrated great specificity, repeatability, and reproducibility [25,26], which is extremely important when addressing the clinical value of CTC biomarkers. The two methods were evaluated in blood samples obtained the same day, thus permitting their comparison in real time. The monitoring of CTCs at different time points during treatment is an additional strength of our study, considering the limited existing data in NSCLC. Nevertheless, the relatively low number of patients and the single-center conduct of the study cannot allow sound conclusions regarding the clinical utility of the two methods and of their combination. Additionally, CTC monitoring during treatment was not assessed at the same time point using the two individual approaches; consequently, their comparison and the evaluation of their complementary prognostic value was feasible at baseline only.

Overall, the results of the current study illustrate that the CellSearch system and the CEACAM5mRNA assay provide comparable CTC detection rates among patients with chemo-naïve locally advanced or metastatic NSCLC, while demonstrating low positivity concordance. Importantly, the two assays provide valuable and complementary prognostic and predictive information, thus implying that their parallel employment may substantially improve patient prognostication. The current study also highlights the importance of CTC monitoring during first line treatment for the real time assessment of disease recurrence in NSCLC. Recent evidence suggests a potential role of serum CEA levels in predicting immunotherapy efficacy in NSCLC patients [32]; thus, it would be of potential interest to investigate the significance of *CEACAM5*mRNA+ CTCs as a biomarker in this patient cohort. Moreover, targeting of CEACAMs holds promising role in the treatment of patients with NSCLC among other cancers [33,34] and a randomized, phase 3 study is already investigating the efficacy of antibody-based targeting of CEACAM5 (SAR408701) to improve PFS and OS in patients with previously treated metastatic NSCLC (NCT04154956). In this context, *CEACAM5*mRNA might be used as a biomarker for the selection of NSCLC patients who would benefit most from therapeutic targeting of CEACAM5.

## 4. Materials and Methods

### 4.1. Patients

The present, single-center study included 43 patients with advanced NSCLC, who received first-line chemotherapy at the Department of Medical Oncology of the University General Hospital of Heraklion (Crete, Greece) from January 2011 to December 2014. Eligible patients had to have histological or cytological confirmation of stage IIIB (not amenable to radical radiotherapy) or IV NSCLC and Eastern Cooperative Oncology Group Performance Status (ECOG-PS) of 0 to 2. Clinical characteristics and follow-up information were prospectively collected.

This study was conducted in accordance with the Declaration of Helsinki ethical guidelines and was approved by the Ethics and Scientific Committees of the University General Hospital of Heraklion, Crete, Greece. All patients gave their written informed consent to participate in the study.

### 4.2. Blood Sampling

CTC analysis was performed by using two different assays, the CellSearch system and the *CEACAM5*mRNA assay. For both assays, PB was obtained at the middle of vein puncture after the first 5 mL were discarded, in order to avoid contamination with epithelial cells from the skin. CellSearch analysis was performed in 43 patients at baseline of chemotherapy, as well as in 22 and 18 patients after the completion of the first and second cycle of treatment, respectively. The *CEACAM5*mRNA assay was performed in 41 patients at baseline and in 25 patients at the end of chemotherapy.

### 4.3. CTC Analysis Using the CellSearch System

Blood (7.5 mL) was collected into CellSave Preservative Tubes, stored at room temperature and processed within 72 h using the semi-automated CellSearch platform (Menarini Silicon Biosystems) for CTC enrichment and enumeration. The reviewer performed the analysis of CTCs without knowledge of the patient’s clinical status. CTC positivity was defined by using two different thresholds: ≥1 CTCs and ≥5 CTCs, as described in previous studies [7,15,16,35].

### 4.4. CTC Analysis Using Real-Time RT-PCR for CEACAM5mRNA

#### 4.4.1. Isolation of PBMCs using Ficoll Density Gradient Centrifugation

Blood (5 mL) was collected in EDTA tubes (Becton Dickinson, Franklin Lakes, NJ, USA) and peripheral blood mononuclear cells (PBMCs) were isolated by Ficoll-Hypaque density gradient (d = 1.077 gr/mol) centrifugation at 650 g for 30 min, as previously described [36,37]. PBMCs were washed twice with phosphate-buffered saline (PBS) and aliquots of 1 × 10^6^ cells were stored at −20 °C until use.

#### 4.4.2. RNA Extraction

Total RNA extraction from PBMCs and the HCC827 cell line was carried out in a laminar flow hood under RNAse-free conditions using Trizol (Thermo Fisher Scientific, Fremont, CA, USA). The isolated RNA was dissolved in RNA storage buffer (Ambion, Austin, TX, USA) and stored at −80 °C until used. Amplification of the *β*-actin, as a house-keeping gene, was performed to verify the RNA integrity, as it has been previously described [25,26].

#### 4.4.3. Quantitative RT-PCR (RT-qPCR)

The reverse transcription and the qPCR conditions have been previously described [25,26]. Quantification of gene expression was performed using the ABI Prism 7900HT Sequence Detection System (Applied Biosystems, Waltham, Massachusetts, USA). All experiments were performed in triplicates. Quantification was based on an external calibration curve that was obtained using external standard cDNAs as previously described [25,26], adapted for NSCLC. cDNA synthesis of HCC827 cell line’s serial dilutions, corresponding to 1–10^5^ cells, was also analyzed in each run. The number of circulating *CEACAM5*mRNA^+^ cells for all of the tested samples was expressed as cell equivalents/5 μg of total RNA. In the current study, the cut-off value of 0.48 cell equivalents/5 μg of total RNA was used to define *CEACAM5*mRNA positivity (cut-off = 3.3 SD/slope, where SD is the standard deviation of the *C*q for 1 HCC827 cell equivalent). 

The SDS 2.3 software was used for the analysis of the results. Finally, genomic DNA contamination was excluded, as no RNA transcripts could be detected in each analyzed sample in the absence of reverse transcriptase.

### 4.5. Statistical Analysis

Chi-square test was used to investigate possible correlations of the status or kinetics of CTCs with patient and disease characteristics. A Wilcoxon test was used to compare CTC counts among different time points. A Kaplan–Meier survival analysis was used to estimate the probability of relapse and death over time. PFS was calculated from the date of the initiation of first-line chemotherapy until the date of disease progression or death from any cause. OS was calculated from the date of the initiation of chemotherapy to death from any cause. The log-rank test was used to compare survival curves between groups. Univariate and multivariate Cox proportional hazard models were used to estimate Hazard Ratios (HR) and the 95% confidence intervals. Multivariate analysis included up to 1 parameter per 10 events. All tests were two-sided, and *p* values were considered significant at the 0.05 level. Statistical analyses were performed using IBM SPSS Statistics version 20.

## Figures and Tables

**Table 1:** Patient and Disease Characteristics (No of patients: *n* = 43).

Parameters	(*n*)	(%)
**Age** (median; 67 years, range; 46–87)		
**Gender**		
Male	39	90.7
Female	4	9.3
**Performance Status**		
0	22	51.16
1	14	32.56
2	7	16.28
**Stage**		
IIIb	8	18.6
IV	35	81.4
**Histological subtype**		
Squamous	14	32.6
Non-squamous	29	67.4
**No of metastatic sites**		
<2	22	51.2
≥2	21	48.8
**Metastatic sites**		
Lung	30	69.8
Bones	16	37.2
Liver	6	14
CNS	6	14
Adrenal gland	8	18.6
Lymph nodes	8	18.6
Peritoneum	1	2.3
Pancreas	1	2.3
**EGFR status**		
wt	41	95.3
mutant	2	4.7
**ALK status**		
wt	43	100
re-arranged	0	0
**Smoking status**		
Non-smoker	6	14
Ex-smoker	12	27.9
Current	25	58.1
**Chemotherapy type**		
Monotherapy	5	11.6
Doublet-chemotherapy	38	88.4
**Best response to treatment**		
Partial response	16	37.2
Stable disease	12	27.9
Progressive disease	11	25.6
Non-evaluable	4	9.3

CNS: central nervous system; EGFR: epidermal growth factor receptor; ALK: anaplastic lymphoma kinase; wt: wild type.

**Table 2:** CTC status of patients using different detection assays (no. of patients: *n* = 41).

CTC Status	CellSearch (≥1 CTCs) *% of Patients*		CellSearch (≥5 CTCs) *% of Patients*	
*CEACAM5*mRNA	Negative	Positive	Negative	Positive
Negative	41.5	29.3	63.4	7.3
Positive	17.1	12.2	26.8	2.4
*p* value (Chi-Square Test)	*p* = 0.986		*p* = 1.000	

**Table 3:** CTC detection using different assays according to best response to first-line chemotherapy.

Detection Method	% of Positive Patients		
CellSearch	PR/SD	PD	*p* value
≥1 CTCs	32.1	63.6	0.146
≥5 CTCs	0	36.4	0.004 *
**Real time RT-PCR**			
CEACAM5mRNA+ CTCs	14.3	60	0.010 *
**Any method**			
*CEACAM5*mRNA+ CTCs and/or ≥1 CTCs	46.4	90	0.025 *
*CEACAM5*mRNA+ CTCs and/or ≥5 CTCs	14.3	90	0.000 *

PR: partial response, SD: stable disease, PD: progressive disease. * Statistical significance at *p* < 0.05; Chi-square test, two-tailed.

## Data Availability

The data presented in this study are available on request from the corresponding author. The data are not publicly available due to ethical restrictions.
